# Multiple Cases of Discoid Menisci within a Family: A Case Report and Literature Review

**DOI:** 10.5334/jbsr.3181

**Published:** 2023-06-30

**Authors:** Thomas Saliba, Paolo Simoni, Alessandro De Leucio

**Affiliations:** 1 ULB, BE; 2 Hopital Universitaire Des Enfants Reine Fabiola, BE

**Keywords:** Discoid meniscus, familial discoid meniscus, heritable discoid meniscus, meniscus anomaly

## Abstract

Discoid menisci are thought to be heritable. However, few documented cases of this occurring within families exist. We present the case of siblings with lateral discoid menisci, documented by knee magnetic resonance imaging (MRI), reinforcing the case for the existence of familial discoid menisci. The children’s father also reportedly had a discoid meniscus, but proof was unavailable due to his country of origin’s poor record keeping. We put this into the context of other rare, reports of similar cases.

**Teaching Point:** We present further case of discoid menisci occurring within families, a long-held belief with little concrete supporting evidence.

## Introduction

The meniscus is a crescent-shaped fibrocartilaginous structure which functions as a shock-absorber, helps joint lubrication, as well as aiding in nutrient and axial load distribution [1]. Menisci appear as low-intensity structures on both T1 and T2-weighted MRI imaging [1]. A discoid meniscus, defined as an enlarged meniscus, extending centrally into the tibio-articular surface, arises in 1 to 5% of knees, being 10–20 times more likely to affect the lateral meniscus [1]. Discoid menisci are more prone to treatment-requiring tears [1].

Discoid menisci have long been thought to be heritable, but few well-documented cases exist, with most being anecdotal and lacking hard proof.

## Case History

**Figure 1:**
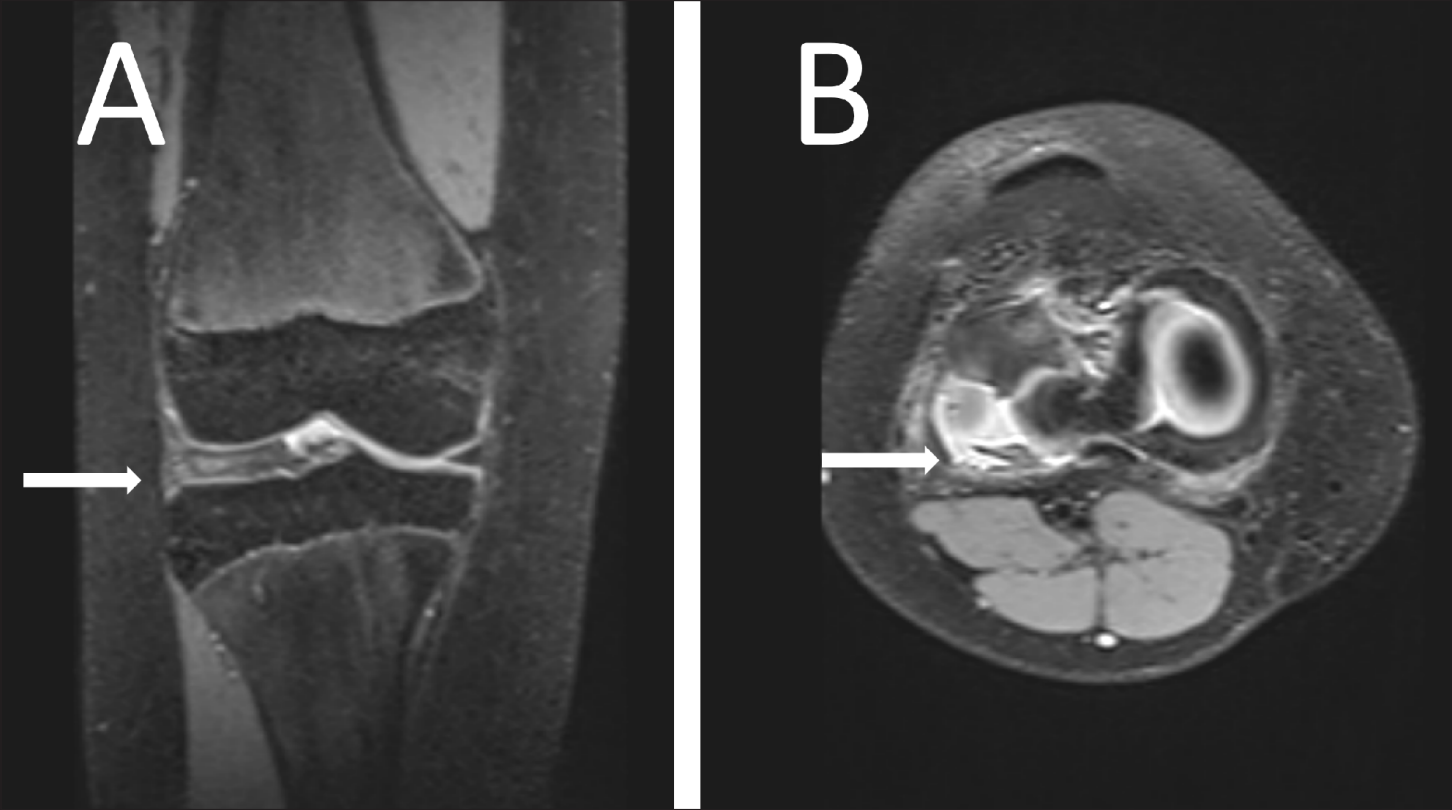
Proton density fat-saturated image of the girl’s knee showing a lateral discoid meniscus (arrow) in a coronal plane **(A)** and a degenerative, disinserted, meniscus with an anterior subluxation and posterior residual meniscal fragment facing the popliteal hiatus (arrow) in an axial plane **(B)**.

**Figure 2:**
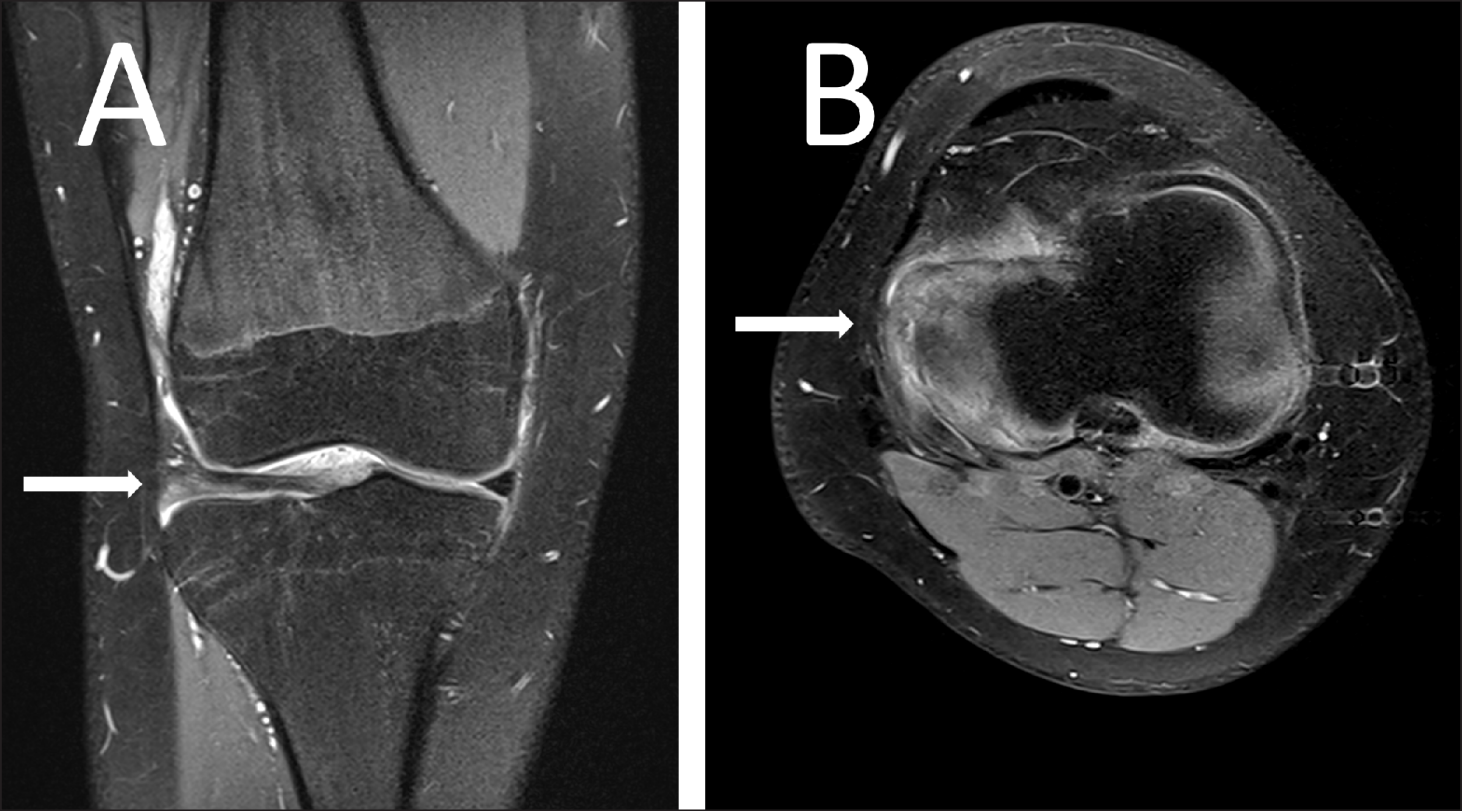
Proton density fat-saturated image of the boy’s knee showing a lateral discoid meniscus (arrow) in a coronal plane **(A)** and a complex posterior horn lesion (arrow) in of the meniscus in an axial plane **(B)**.

A boy and girl of north-African origin, born of the same non-consanguineous parents, presented to orthopaedist complaining of recurrent knee pain and swelling. The girl underwent a knee X-ray and ultrasound, both were unremarkable. A follow-up magnetic resonance imaging (MRI) was ordered, revealing a degenerative and subluxated lateral discoid meniscus, determined to be causing her ailment (Figure 1). Her brother also underwent a knee MRI and was found to have a discoid meniscus, with a posterior horn lesion alongside an anterolateral ligament complex and popliteal ligament injury (Figure 2). When the father was questioned, he claimed have also had a discoid meniscus, though his medical records were lost in his country of origin, making verification impossible. Both siblings underwent surgery for their discoid menisci, leading to an uneventful recovery.

## Comments

Although theories about the origin of discoid menisci abound, the precise origins are unknown. Smillie suggests the discoid shape may be a foetal intermediate stage, failing to progress into a mature state, though embryological evidence is lacking [2,3]. Kaplan theorises that it originates from a deficient posterior meniscal attachment, resulting in hypermobility and associated micro-traumatisms, causing morphological changes [4]. However, this theory doesn’t explain cases with normal posterior meniscal attachments [3]. The third theory is that discoid meniscuses have a hereditary component yet proven familial cases are rare [3]. A literature search revealed only five other reported cases of families with multiple members presenting with discoid menisci. One case included two brothers in whom discoid menisci were found following MRIs for knee pain, the mother also claiming to have had a discoid meniscus discovered after arthroscopy, though her claim is based on her self-reported history and drawing by her surgeon [5]. Another report, from France, found bilateral lateral discoid menisci in three brothers and one sister but not their parents [6]. Yet another report from the USA describes a family in which discoid menisci were discovered in a brother and sister as well as their father, with arthroscopic confirmation [7]. A further case from the USA reported bilateral lateral discoid menisci in identical twin sisters, with X-rays and arthrograms performed, confirmed by arthrotomy [8]. The father was also symptomatic, but his arthrogram was negative [8]. Finally, there exists a series of Chinese reports, finding six cases of two generations having arthroscopically confirmed discoid menisci [9].

We presented a case of lateral discoid menisci in siblings with MRI evidence, and anecdotal evidence of a discoid meniscus in the case of their father. Our case is unique due to the high quality of the imagery, not found in the other cases, providing proof of the diagnosis. We add further solid evidence for the heritability of discoid menisci.

## Conclusion

Discoid menisci are rare, occurring preferentially in the lateral meniscus [1]. They are believed to have a heritable component, but few documented cases exist. We provide a clear-cut example of siblings with lateral discoid menisci, with anecdotal evidence in the case of their father. We provide further evidence that discoid menisci are heritable, supporting this long-held belief.

## References

[1] Nguyen JC, de Smet AA, Graf BK, Rosas HG. MR imaging–based diagnosis and classification of meniscal tears. Radiological Society of North America. 2014; 34: 981–99. DOI: 10.1148/rg.344125202 25019436

[2] Smillie I. The congenital discoid meniscus. J Bone Joint Surg Br. 1948; 30: 671–82. https://pubmed.ncbi.nlm.nih.gov/18894619/. DOI: 10.1302/0301-620X.30B4.671 18894619

[3] Sun Y, Jiang Q. Review of discoid meniscus. Orthop Surg. 2011; 3: 219–23. DOI: 10.1111/j.1757-7861.2011.00148.x 22021136 PMC6583433

[4] Kaplan E. Discoid lateral meniscus of the knee joint: Nature, mechanism, and operative treatment. J Bone Joint Surg Am. 1957; 39: 77–87. https://pubmed.ncbi.nlm.nih.gov/13385265/. DOI: 10.2106/00004623-195739010-00008 13385265

[5] Ahmed Ali R, McKay S. Familial discoid medial meniscus tear in three members of a family: A case report and review of literature. Case Rep Orthop. 2014; 2014: 1–5. DOI: 10.1155/2014/285675 PMC4274651 25548700

[6] de Lambilly C, Pascarel X, Chauvet J, Marle J, Honton J. External discoid menisci. Apropos of a familial series of 6 cases. Rev Chir Orthop Reparatrice Appar Mot. 1991; 77: 359–61. https://pubmed.ncbi.nlm.nih.gov/1836889/. 1836889

[7] Dashefsky J. Discoid lateral meniscus in three members of a family. J Bone Joint Surg. 1971; 53: 1208–10. https://www.jbjs.org/reader.php?id=12181&rsuite_id=347699&source=The_Journal_of_Bone_and_Joint_Surgery/53/6/1208/abstract&topics=kn%2Bpd#info. DOI: 10.2106/00004623-197153060-00018 5109802

[8] Gebhardt MC, Rosenthal RK. Bilateral lateral discoid meniscus in identical twins. J Bone Joint Surg. 1979; 61: 1110–1. https://journals.lww.com/jbjsjournal/Citation/1979/61070/Bilateral_lateral_discoid_meniscus_in_identical.27.aspx. DOI: 10.2106/00004623-197961070-00027 582824

[9] Wen H, Zhang Y, Liu Z. Lateral discoid meniscus of the knee in two consecutive generations. Chinese Journal of Minimally Invasive Surgery. 2001; 12. https://pesquisa.bvsalud.org/portal/resource/pt/wpr-584303.

